# Preventive and therapeutic effect of vitamin D on depression-like behavior in a mouse adolescent depression model and its association with BDNF protein expression

**DOI:** 10.3389/fpsyt.2024.1425681

**Published:** 2024-07-29

**Authors:** Xueping Yang, Junxiao Miao, Yinglin Huang, Lili Li, Gengsen Zhuang

**Affiliations:** ^1^ Department of Psychology, The People’s Hospital of Liaoning Province, The people’s hospital of China Medical University, Shenyang, Liaoning, China; ^2^ Department of Psychiatry, Sheng Jing Hospital of China Medical University, Shenyang, Liaoning, China; ^3^ Department of Psychiatry, The First Affiliated Hospital of China Medical University, Shenyang, Liaoning, China; ^4^ Department of Psychiatry and Mental Health, The Medical University of Dalian, Dalian, Liaoning, China

**Keywords:** depression, vitamin D, BDNF, depression model, mouse

## Abstract

**Introduction:**

Previous studies in different populations have shown that vitamin D supplementation may reduce depression levels. In adolescents, vitamin D deficiency has been identified as a factor contributing to the onset of depression. This study aimed to establish a model of adolescent depression in mice by using the scientific unpredictable chronic mild stress (UCMS) model and to preliminarily evaluate the effect of vitamin D on the occurrence and development of depression and whether it is related to the protein expression of the BDNF pathway.

**Methods:**

The UCMS method was used to establish a model of adolescent depression in 4-week-old C57BL/6 male mice, randomly divided into five groups: Control group, Stress group, Stress+ low-dose group, Stress+ medium-dose group, Stress+ high-dose group. At the same time as chronic stress, the administration groups were given intramuscular injections of different doses of vitamin D. After 8 weeks, behavioral tests, including the forced swimming test (FST) and open field test (OFT), were performed on each group of mice, along with recording of indicators, blood vitamin D level detection, and brain tissue western blot analysis.

**Results:**

The results showed a significant difference in vitamin D levels among mice in different groups after 8 weeks (P=0.012). The results of behavioral testing showed a significant difference in the static time of forced swimming among the groups (P<0.001). Compared with the UCMS group, the static time of mice with vitamin D injection was significantly reduced (P<0.001). The total number of times mice entered the central area, the total distance of movement, and the time spent in the central area significantly increased after vitamin D injection compared with the UCMS-only group (all P<0.001). There was no significant difference in the expression of BDNF in the brain tissues of experimental mice (P>0.05).

**Discussion:**

In conclusion, in the mouse adolescent depression model, appropriate vitamin D supplementation can reduce the occurrence of stress-induced depression. Furthermore, vitamin D deficiency may also serve as a potential risk factor for depression.

## Background

Adolescence is an important period of psychological development, a stage of continuous growth and dynamic change. Physiological, psychosocial, and cognitive changes make adolescents susceptible to psychological disorders. Depression is a psychological disorder with a high incidence and is the leading cause of disability worldwide. A meta-analysis showed that the global point prevalence of depression was 34% between 2001 and 2020 based on self-reported depressive symptoms. The point prevalence of major depressive disorder (MDD) and dysthymia was 8% and 4%, respectively. Furthermore, depressive symptoms in adolescents increased from 24% between 2001 and 2010 to 37% between 2011 and 2020. The Middle East, Africa, and Asia have the highest prevalence of elevated depressive symptoms (1).

Since depression typically begins in adolescence, juvenile cases are more likely to be first cases, while adult cases may be relapses from earlier juvenile cases. Symptomatic characteristics differ between adults and adolescents. Autonomic dysregulation symptoms (insomnia, fatigue, and changes in appetite and weight) are more common in adolescents with MDD than in adults. Further, anhedonia/loss of interest and concentration problems are more common in adults with MDD. The differences in the presentation of depression in adolescents and adults may be related to different pathophysiological mechanisms. In addition to targeting those who already have clinical depression, research and policy should focus on education and supportive efforts to prevent adolescents from developing depression (2). It is important to study the psychological state of adolescence, and some factors and behaviors related to adolescence, such as poor school performance, may be associated with depression in adulthood, so early detection and management of externalizing disorders in children and adolescents is important (3). It is necessary to pay attention to early depressive symptoms, and more research data—especially on the influencing factors and mechanisms of depressive symptoms—are needed to support the prevention and treatment of depression in adolescents.

Research on factors related to depression has shown the benefits of vitamin D in the prevention and treatment of depression. Vitamin D deficiency (VDD) may be an associated risk factor for depression. VDD has a potential causative role in depression and suicide, while vitamin D supplementation has shown potential benefits in the adjunctive treatment of mood disorders, helping to reduce the risk of depression (4). Although some study results have been inconsistent, meta-analyses investigating the efficacy of vitamin D supplementation and the relationship between vitamin D and depression showed that vitamin D can significantly reduce depressive symptoms. People with lower serum vitamin D levels were found to have higher rates of depression, and vitamin D supplementation and increasing serum vitamin D levels had potential benefits in reducing the development and symptoms of depression (5). However, more extensive and detailed research on adolescent depression and vitamin D is needed.

To date, clinical studies on the therapeutic effect of vitamin D have paid more attention to the improvement of depressive symptoms in the elderly, among whom depressive symptoms and impaired physical function are very common. Studies have found that vitamin D supplementation can improve both of these conditions, especially for people with low vitamin D levels (6, 7). The cross-sectional data statistical analysis found that vitamin D level was inversely associated with depression, and both vitamin D deficiency and older age were associated with a higher risk of depression (8). Through double-blind randomized clinical trial, and serum vitamin D and depression severity evaluation, it was found indeed that the severity of depression of the intervention group (50000IU cholecalciferol/2 weeks) improved with an increased level of vitamin D for adult patients with depression, compared to the placebo-control group. However, the long-term effects of vitamin D on depression were not evaluated, and the way by which the vitamin D mediate depression was not explained (9). However, some studies have not found vitamin D to be therapeutic for depression in adults. In one outpatient multicenter study conducted between 2010 and 2013, patients aged 18–65 diagnosed with mild to severe depressive disorder were randomly assigned to receive either vitamin D supplements or placebo. However, there was no significant reduction in depressive symptoms on the Hamilton scale after vitamin D supplementation compared with placebo (10). However, long-term follow-up controlled clinical trial also found no significant difference in the risk of depression or clinically related depressive symptoms between the intervention and placebo groups after vitamin D supplementation, and the results did not support preventing effect for depression (11). Therefore, the results of studies related to vitamin D and depression are not completely consistent.

In the adolescent population, it is thought that lower vitamin D levels may be associated with depression. Vitamin D deficiency has been found to play a role in the onset of depression (12). The results of a study based on the detection of blood 25(OH)D levels and self-reported depressive symptoms in Chinese adolescents explored the cross-sectional and longitudinal association between vitamin D and depression in early adolescence. Higher baseline serum 25(OH)D levels were found to be associated with a higher risk of depressive events, while baseline VDD was associated with an increased risk of depression. The results also support the potential beneficial role of vitamin D supplementation in reducing the risk of depression in early adolescence (13). Overall, there is growing evidence that vitamin D may affect mental health in addition to its association with calcium and phosphorus homeostasis and bone health. However, assessing vitamin D status in adolescents with depression or supplementing with vitamin D is not currently part of routine treatment.

Controlled intervention studies are essential to demonstrate whether vitamin D is associated with improving depressive symptoms in adolescents. A study on VDD patients with psychiatric disorders hospitalized in a psychiatric department confirmed that patients who received daily vitamin D supplementation showed improvements compared those who did not receive daily vitamin D supplementation (14). To prevent the risk of overdose, vitamin D supplements still need to be closely monitored, and safer ways are still needed for the population. However, for adolescent depression, due to the specificity of the population, relevant studies in animal models may still be a safer and more reliable research method, and vitamin D has shown a significant effect on the treatment of depression in successfully established animal models (15). It is clear that more human studies and animal models are needed to deepen understanding of the biological link between vitamin D and depression, to advance understanding of the various pathogeneses and pharmacological therapies of the disorder, and to increase the potential for new prevention and treatment options for adolescent depression.

The chronic mild stress (CMS) model is a widely used model for studies such as those on the effects of antidepressants. It was first developed nearly 40 years ago, based on the observation that long-term exposure to unpredictable/uncontrollable mild stressors in rodents can lead to reduced palatable fluid intake, behavioral hopelessness, motor inhibition, anxiety-like changes, and nutritional (somatic) abnormalities. Different studies have adapted this model to suit different needs (16). For animal models, positive effects of vitamin D on neuroprotection in the hippocampus, which plays a role in alleviating depression-like symptoms, have been found in adult mice (15).

The unpredictable chronic mild stress (UCMS) model is a commonly used depression model in depression-related research. Although originally designed for rats, the model is now also used in mice and is a very valuable model for gaining insight into the etiology and developmental components of major depressive disorder as well as identifying new therapies (17). The behavioral responses associated with different stress patterns are quite complex. The UCMS model has some advantages over the CMS model, and studies have examined the effects of two chronic stress regimens on anxiety-like and depressive behaviors. After 4 weeks of unpredictable chronic mild stress or chronic restraint stress, both models of chronic stress were found to produce anxiety-like behaviors, but only unpredictable chronic mild stress could induce depressive behaviors (18). However, there is still a lack of relevant research on the establishment of animal models of adolescent depression and the effect of vitamin D on the occurrence and development of depression in animal models.

Previous animal experiments have shown that different substances and pathways may improve or aggravate depressive symptoms, which may be related to the brain-derived neurotrophic factor (BDNF) pathway. As a result, BDNF has emerged as an important determinant of antidepressant efficacy (19). There is also considerable research on how depression-like behaviors can be produced by the BDNF signaling pathway, and some studies have shown that chronic stress exposure can induce the development of depression-like behavior though damaging the signaling between cyclic adenosine monophosphate-response element binding protein (CREB) and BDNF in the hippocampus, although the underlying mechanism remains largely unknown. Given the critical role of impaired CREB-BDNF signaling in depression, chronic unpredictable stress may significantly increase hippocampal NR6A1 protein expression levels, decreasing hippocampal CREB phosphorylation and BDNF protein expression. This leads to the occurrence of depression-like behaviors by impairing the CREB-BDNF signaling cascade (20). Therefore, the effect of VDD on the occurrence of depression or the improvement of depressive symptoms by vitamin D may also occur through the BDNF signaling pathway.

For the relevant animal studies of vitamin D, some studies have explored the antidepressant effects of vitamin D by using mouse animal studies. To discover the preventive and therapeutic effects of vitamin D on the depressive behaviors, control studies were manipulated by drug induction and depressive behaviors were evaluated by behavioral testing (21). Combined with the above studies, we hypothesized that vitamin D has some preventive effects on the development of depression, assuming the existence of factors that induce depression such as chronic mild stress. If the intervention is done by vitamin D supplementation, the depressive outcome may be altered even in the presence of stress factors. Details of vitamin D supplementation can be clarified further through the serum vitamin D detection, and the relationship between vitamin D and depression can be illustrated better (22). If vitamin D plays a preventive role on depression, it may be through the nerve loop. Therefore, in this study, we established a mouse model of adolescent depression through the scientific UCMS model and provided long-term supplementation of different doses of vitamin D in the form of experimental control in order to investigate the preventive and therapeutic effects of vitamin D in depression, preliminarily evaluate the role of vitamin D in the development of depression, determine whether this role is related to the protein expression of the BDNF pathway through western blot detection, and obtain corresponding data to lay the foundation and provide support for in-depth research in larger samples in the future. Research method by non-oral administration-vitamin D injection and vitamin D detection in the blood was more accurate for vitamin D dose and related effects than previous studies.

## Method

### Materials

#### Animals

C57BL/6J mice, 4 weeks old, male, SPF-grade, were provided by Sipeifu Animal Company. All mice were randomly divided into five groups, 15 ones in each group. This study was approved by the animal ethics review board of China Medical University.

#### Main experimental agents

The main materials used were vitamin D3 (cholecalciferol, Solarbio), a mouse 1,25 dihydroxyvitamin D3 (1,25-(OH)2D3) enzyme-linked immunoassay kit (96T) (Jiangsu Enzyme Immunoassay Co., Ltd., MEIMIAN), antibody (target BDNF, working concentration 1:1,000; actin, working concentration 1:5,000), secondary antibody (goat anti-rabbit IgG[H+L]/HRP, final concentration 1:10,000) (Jackson), and sample diluent. Others included lotion, TBST, blocking solution, antibody diluent, electrophoresis solution, sample loading buffer, electroporation solution, a membrane (PVDF membrane pore size 0.45 μm, Millipore), filter paper (3 mm), and a chemiluminescence imaging system.

#### Main experimental equipment

The experimental equipment included a microplate reader (352 type, Labsystems Multiskan), plate washer (AC8 type, Thermo Labsystems), micro-high-speed centrifuge (instrument model: TG16W type, Xiangyi Group), water-isolated constant temperature incubator (GNP-9080 type, Shanghai Jinghong), electrophoresis instrument (Mini-PROTEAN Tetra Cell, Bio-Rad), wet transfer instrument (PowerPac Basic, Bio-Rad), chemiluminescence imaging system (FluorChem E ProteinSimple), multichannel pipette (300 μL Thermo), and vortex shaker (LP Vortex Mixer, Thermo).

### Experimental methods

#### Establishment of animal models of adolescent depression and vitamin D3 administration

The UCMS method was used to establish an animal model of adolescent depression (17, 18), and various stimuli, such as tail clamping for 2 min, fasting for 24 h, water fasting for 24 h, wet bedding for 24 h, falling from heights, strange objects (multicolored building blocks/plastic cups), and odors (alcohol), were used. According to the experimental standard strictly, the experimental conditions and time were controlled strictly, ensuring that the stress and operation conditions of the groups were consistent. Male mice were randomly divided into five groups: Control group (normal saline injection alone), Stress group (UCMS and normal saline injection), Stress+ low-dose group, Stress+ medium-dose group, and Stress+ high-dose group (UCMS and injection of different doses of vitamin D3). All the stress groups were randomly modeled with two to three stimuli per day without recurrence for 8 weeks. Stress was performed at the beginning of the experiment. To observe the long-term effects of vitamin D on mice, it was started at an earlier time, and the duration of stress was 8 weeks instead of 4 weeks. At the same time, at the onset of stress, the stress + vitamin D groups received intramuscular injections of diluted vitamin D3, with 400 IU/week/mouse in the low-dose group, 800 IU/week/mouse in the medium-dose group, and 1,600 IU/week/mouse in the high-dose group, and normal saline injections were administered into the Control group and the Stress group simultaneously. Stress and the injection of vitamin D3 lasted for 8 weeks. Behavioral testing was performed one week after finishing injection.

#### Animal behavior testing

##### Forced swimming test

The principle of the forced swimming experiment is that the mouse is placed in a small container of water, and when, after repeated struggles, it cannot escape, the mouse will become stationary and keep itself floating on the surface of the water with a small limb movement, which is considered to be immobile time. The specific method employed involved filling a 2-L beaker with 22–25°C warm water to a 12-cm height. Subsequently, the mouse was placed in the beaker and a timer was set for 6 min. The behavior of the mouse was then tracked and analyzed using video tracking software (Smart 3.0), with the immobility time, which was within the last 4 minutes, being recorded.

##### Open field test

The open field test, also known as the open box test, is a method of evaluating spontaneous activity, exploratory behavior, and spontaneous anxiety in rodents. The open box is a 40-cm-long × 40-cm-wide × 40-cm-high observation box with an opening and white walls and surfaces, and the bottom of the box is divided into the central area and the surrounding area. A single mouse was placed in the central area of the open box, and each mouse was observed for 5 min. The experiment was carried out in a quiet room, and the top of the open box was equipped with a camera to observe and record the mouse’s behavior. The behavioral video of the mice was tracked and analyzed using video tracking software (Smart 3.0). Before each mouse was tested, the inner walls and bottom surface of the open box were cleaned with 75% ethanol to prevent any residual information from the last animal (such as the animal’s stool, urine, or smell) from affecting the results of the next test. The OFT recorded metrics were total distance of movement, total number of entries into the central area, and time spent in the central area.

##### Material acquisition

When the animals were grouped, apical blood from the tail tip was collected. After the behavioral experiment, the animals were sacrificed. Blood from the mouse eyeballs was taken in an EP tube, left at room temperature for 60 min, and centrifuged at 3,000 r/min for 15 min at room temperature; the supernatant was stored in a −80°C refrigerator for later use. After the blood collection was completed, the experimental mice were decapitated, the brain was removed, and the hippocampus was separated on ice in an RNase-free EP tube and transferred to a −80°C freezer for later use.

##### Western blot BDNF protein level analysis

To analysis the BDNF protein level, hippocampus tissue was performed by Western blot. The main steps of the western blot analysis were as follows: (1) Loading: The processed tissue sample was directly loaded with a sample volume of 30 μg. (2) Electrophoresis: A constant voltage of 100 V was applied until the bottom of the glue plate of bromophenol blue was running. (3) Film transfer: A constant pressure of 110 V was applied for 90 min. (4) Closure: Closure was performed at 4°C overnight. (5) Primary antibody incubation: The antibody was diluted and incubated at room temperature for 2 h. (6) Elution: A destaining shaker was used to elute the membrane 3 times for 5 min each time. (7) Secondary antibody incubation: The secondary antibody was diluted and incubated at room temperature for 2 h. (8) Elution: A destaining shaker was used to elute the membrane 3 times for 5 min each time. (9) Color development: A chemiluminescence imaging system was used for color development. (10) BCA: This included the preparation of standards, preparation of analytical samples, spotting, color development, and reading.

##### BDNF protein level detection

Gel image analysis was performed, the film was scanned, and the net optical density value of the target band was analyzed using AlphaEaseFC and statistically analyzed.

##### Statistical analysis

SPSS 22.0 (version 22.0, SPSS Inc., Chicago, IL, USA) was used for statistical processing. All data were presented as mean ± standard deviation (mean ± SM). Single-factor ANOVA, *t*-tests, and Chi-square tests were used for between-group comparisons and multi-group comparisons, with *P*<0.05 indicating statistical significance.

## Results

### Changes in blood vitamin D levels in each group

**Figure 1:**
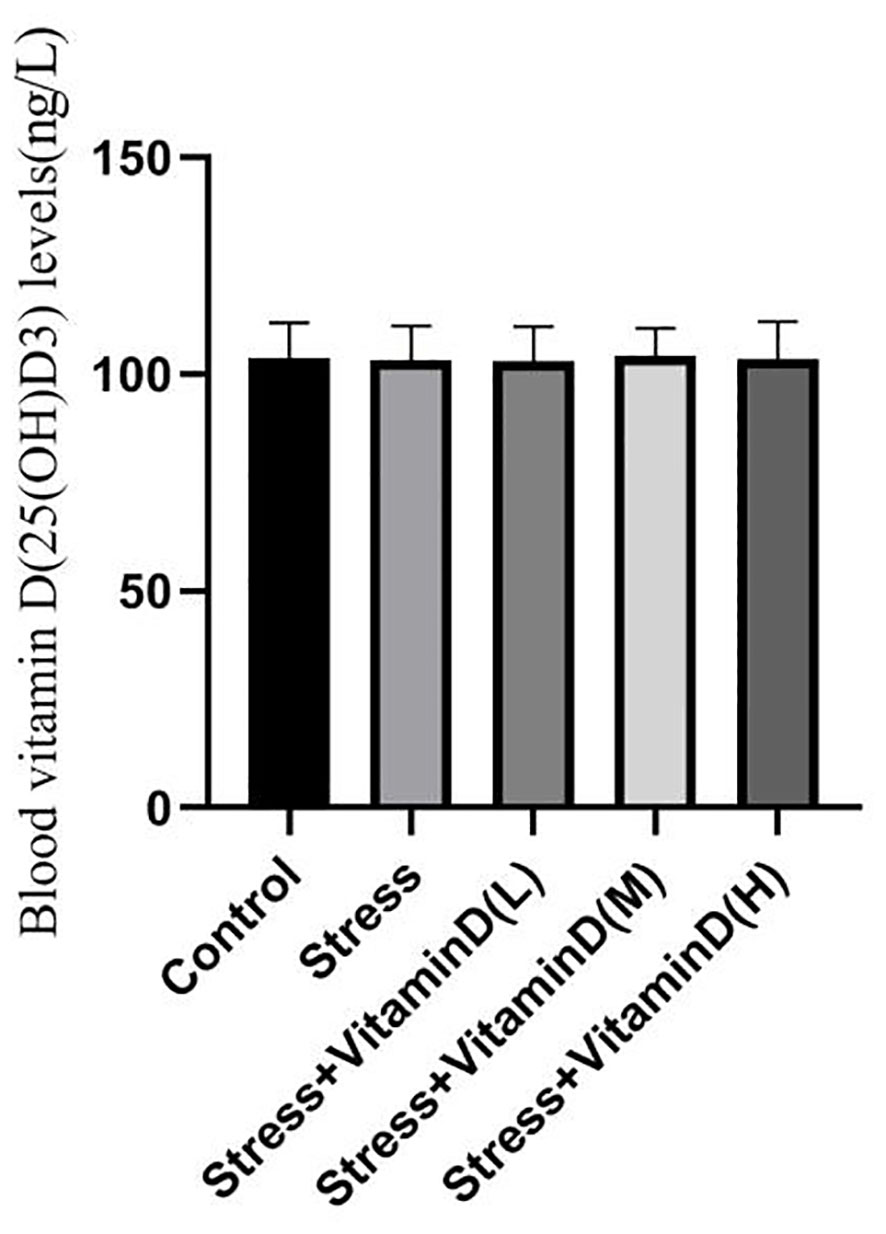
Comparison of the baseline blood vitamin D(25(OH)D3) levels in each group mice.

**Figure 2:**
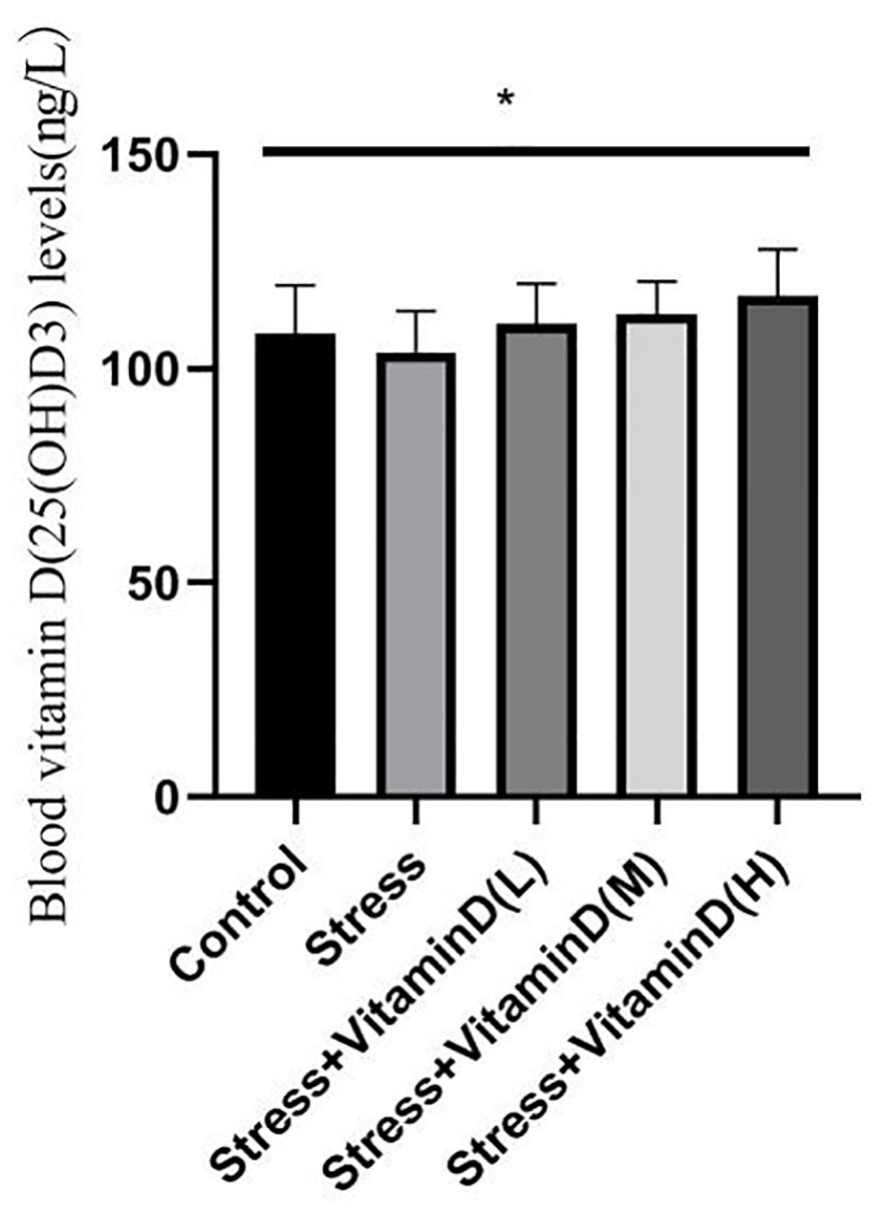
Comparison of the blood vitamin D(25(OH)D3) levels in each group mice after 8 weeks. *p<0.05.

As shown in **Figures 1** and **2**, at the baseline level, there was no significant difference in vitamin D levels among groups (K=0.068, P=0.999). After 8 weeks, there was a significant difference in vitamin D levels among groups (K=12.806, P=0.012). There was no significant difference between the control group and the UCMS alone group (K=1.389, P=0.239). There were, however, significant differences between the low-dose group and the control and UCMS alone groups (K=12.806, P=0.012), between the medium-dose group and the control and UCMS alone groups (K=6.781, P=0.034), and between the high-dose group and the control and UCMS alone groups (K=9.585, P=0.008). The results showed that the 1,25(OH)D3 blood levels in mice were indeed increased by vitamin D injection, but there was no significant difference among the three vitamin D injection groups (K=2.657, P=0.265).

### Effect of vitamin D on forced swimming static time in a depressive model of UCMS mice

**Figure 3:**
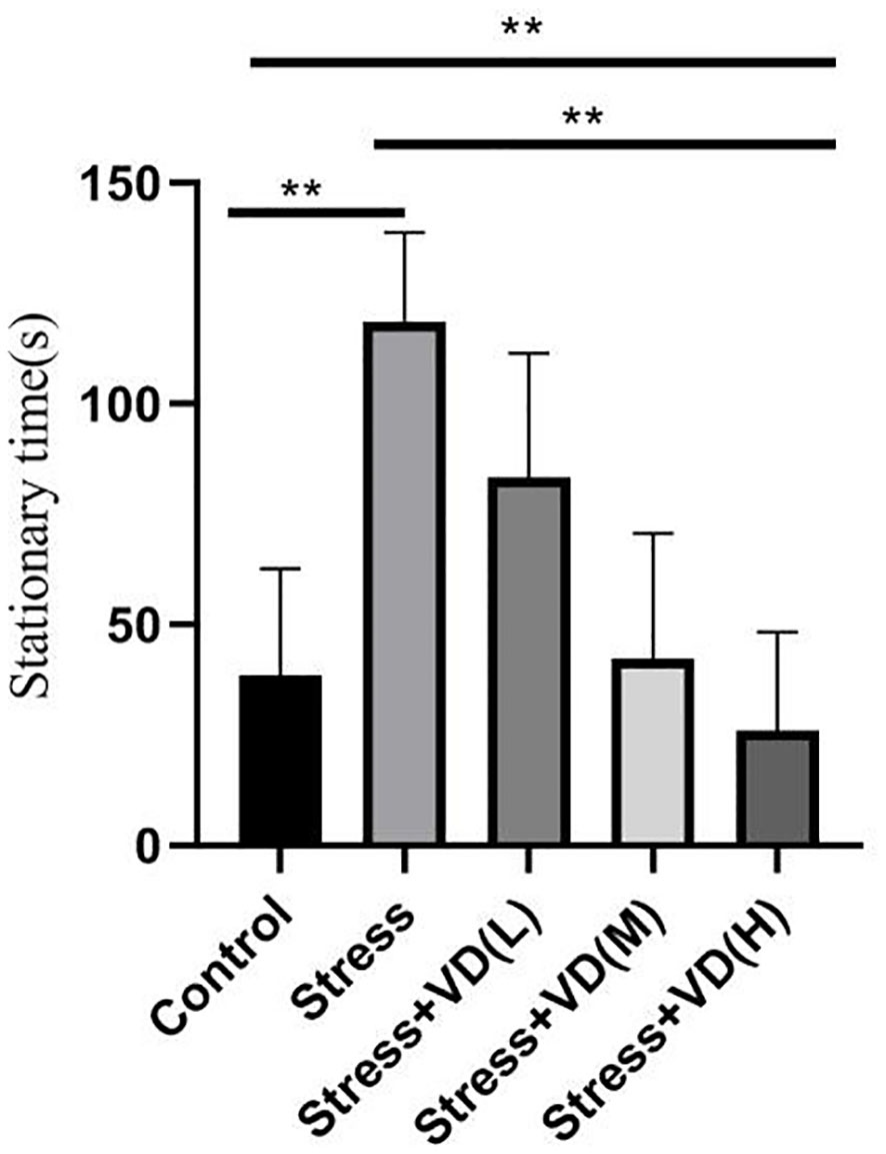
Comparison of the stationary time of the forced swimming test in each group mice. **p<0.01.

As shown in **Figure 3**, there were significant differences in forced swimming test results among the groups (K=45.703, P<0.001). The stationary time of the mice with UCMS alone was significantly higher than that of the control group (K=19.864, P<0.001), but the stationary time of UCMS mice after different doses of vitamin D injection was significantly lower than that of the mice with UCMS alone (K=39.836, P<0.001); the difference was extremely significant, and the reduction was the smallest in the low-dose vitamin D group.

### Effect of vitamin D on the total number of entries into the central zone by UCMS mice in the open field test

**Figure 4:**
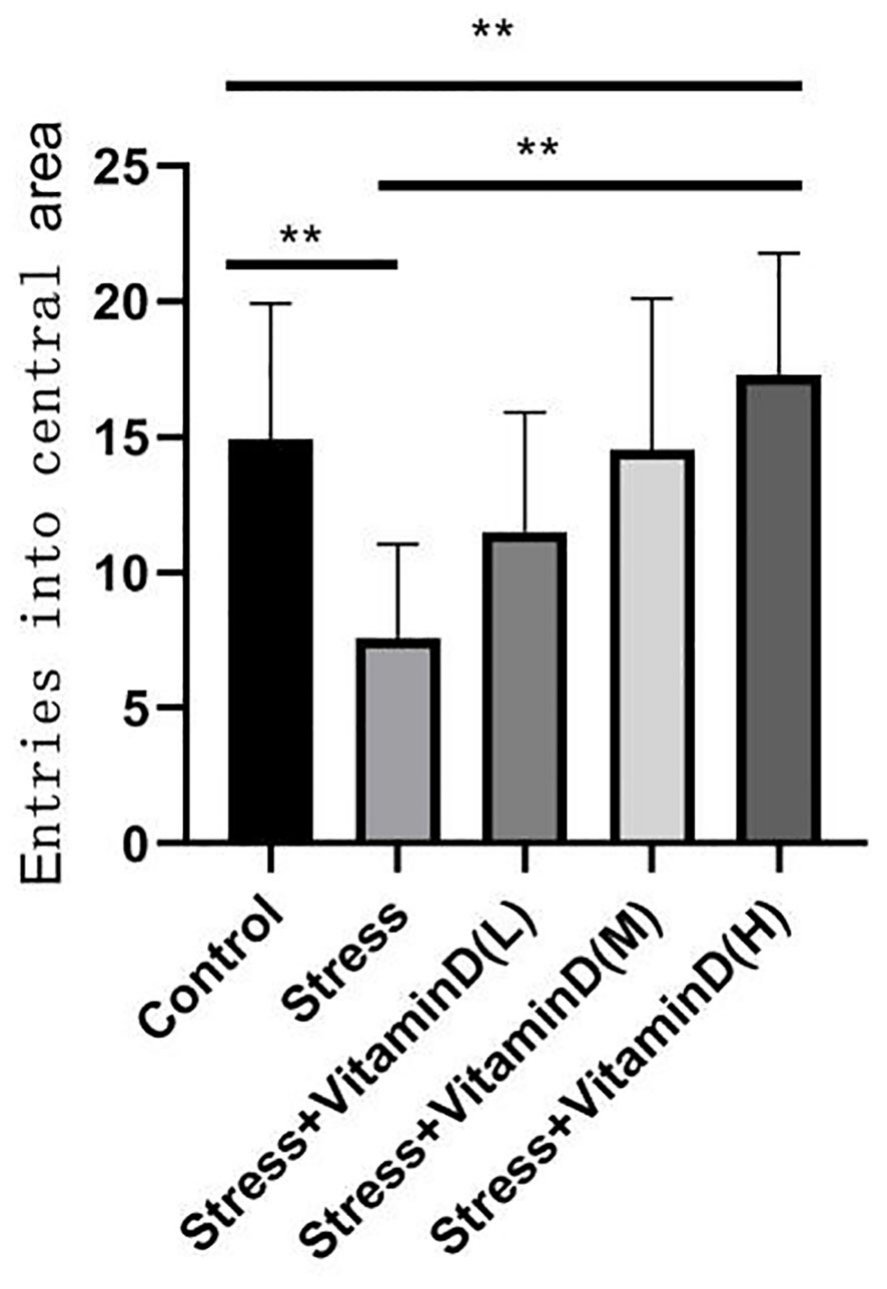
Comparison of the total number of entries into the central area by each group mice. **p<0.01.

**Figure 5:**
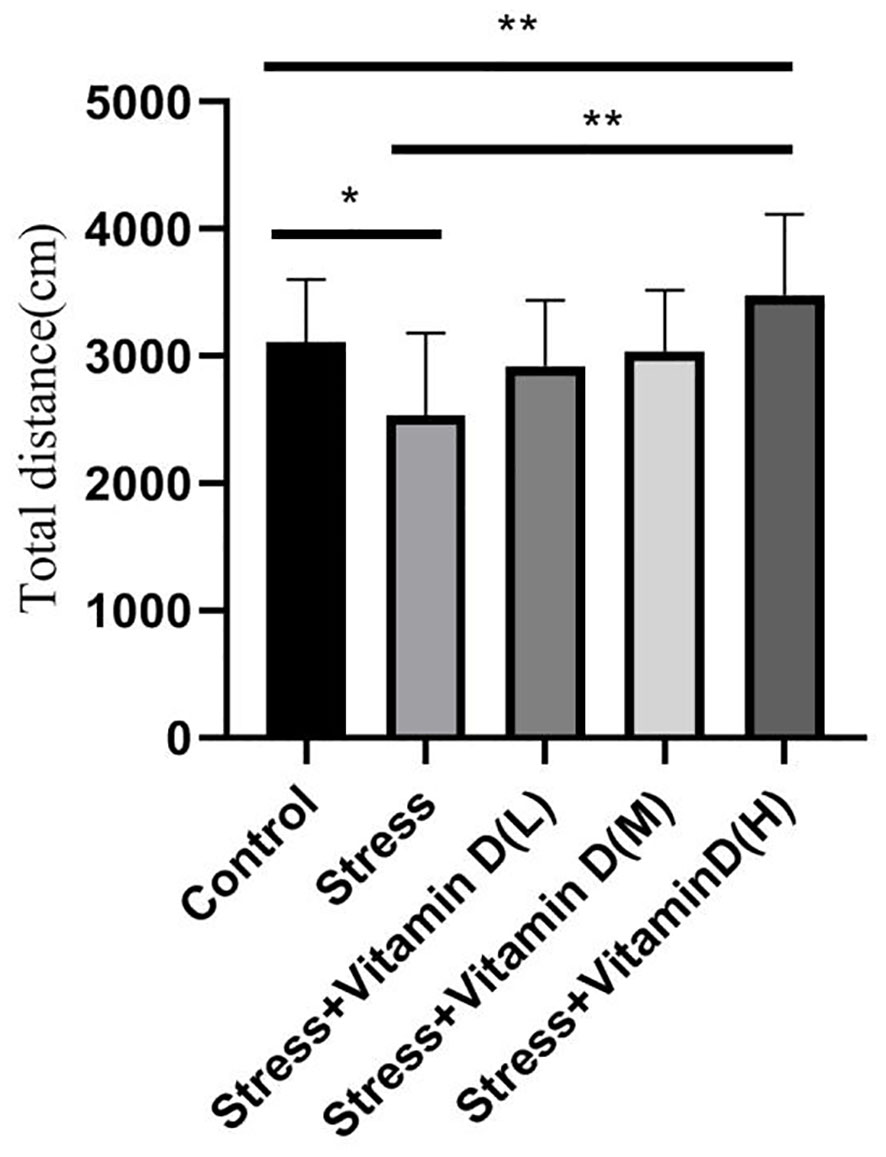
Comparison of the total moving distance of each group mice. *p<0.05, **p<0.01.

**Figure 6:**
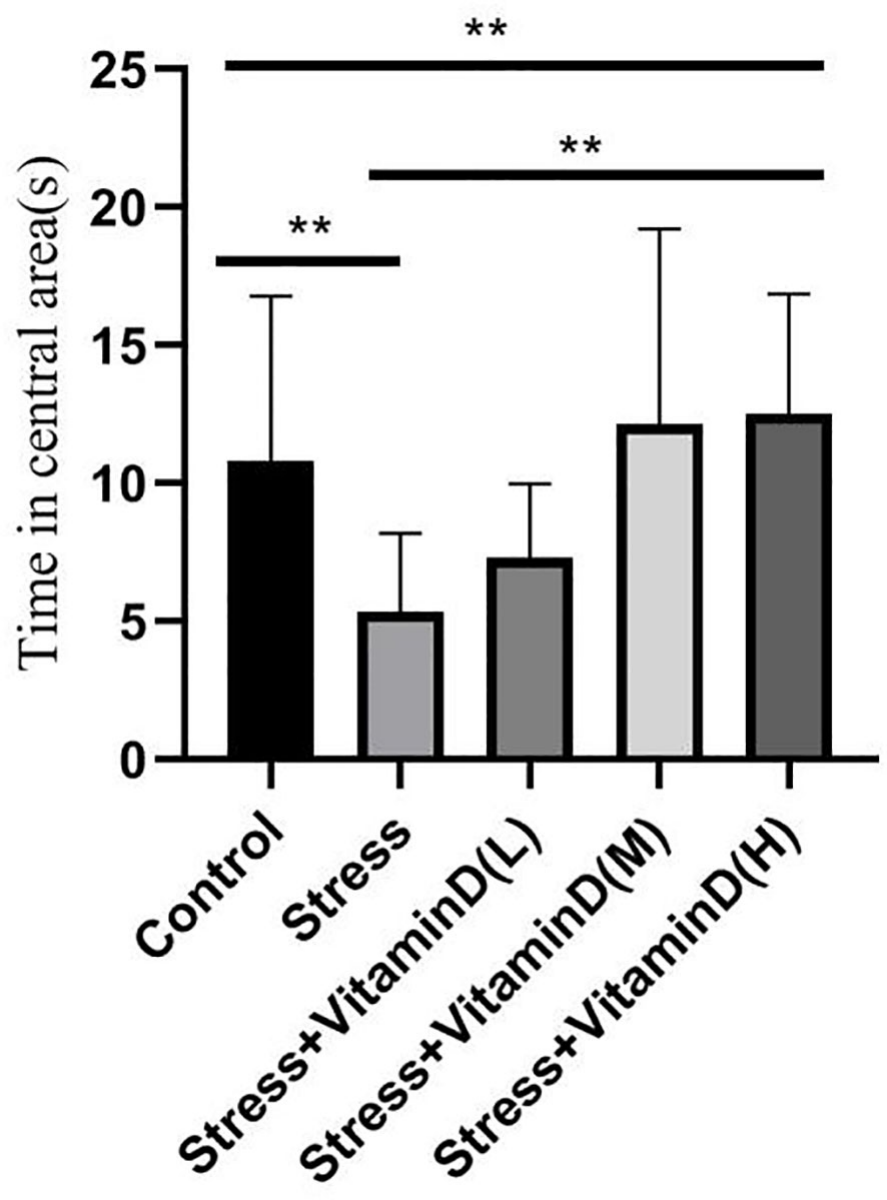
Comparison of the time spent in the central area by each group mice. **p<0.01.

The results of the open field test showed a significant difference among groups (K=26.351, P<0.001), with the total number of entries into the central area being significantly lower for UCMS mice than for the control group (K=13.012, P<0.001), but the total number of entries of UCMS mice after different doses of vitamin D injection was significantly higher than that of mice with UCMS alone (K=23.610, P<0.001) (**Figure 4**). For the total moving distance of mice, the analysis results showed significant differences among the groups (K=14.623, P=0.006), and the total distance of mice with UCMS alone was significantly lower than that of the control group (K=5.709, P=0.017), but the total distance of UCMS mice after different doses of vitamin D injection was significantly higher than that of mice with UCMS alone (K=13.582, P=0.004) (**Figure 5**). For the time spent in the central area, there were also significant differences among groups (K=26.321, P<0.001). The time of UCMS mice with UCMS alone was significantly shorter than that of the control group (K=11.567, P=0.001), but the time of UCMS mice after different doses of vitamin D injection was significantly longer than that of mice with UCMS alone (K=23.890, P<0.001) (**Figure 6**). The behavioral testing indicated that UCMS alone and the control group were significantly different (*p*<0.05), indicating that the depression model was successfully established.

### Comparison of BDNF expression levels in the brain tissues of each group of mice

**Figure 7:**
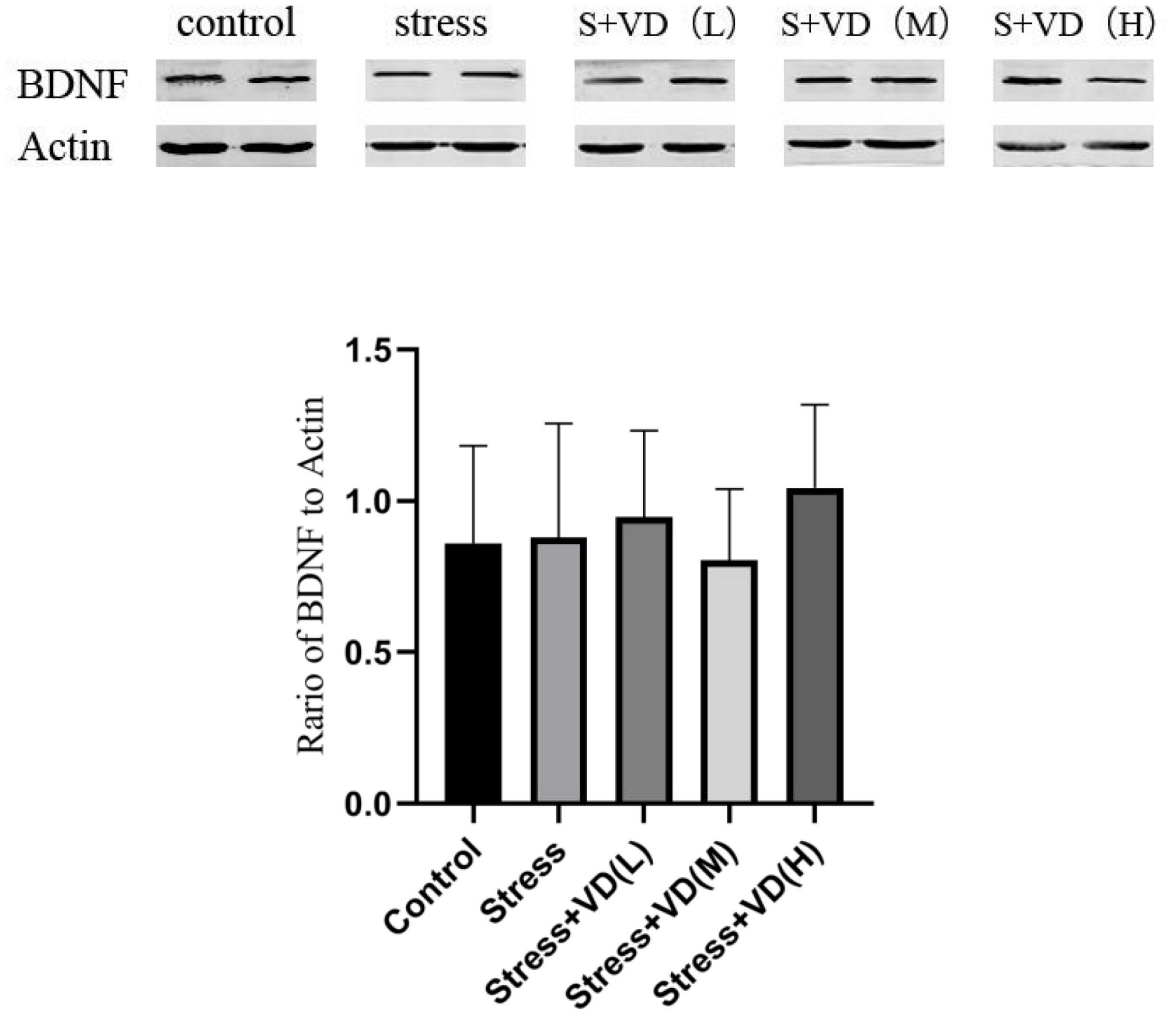
Comparison of BDNF expression levels in brain tissues of each group of mice.

The comparison of BDNF expression among groups was carried out by analysis of the band results of the western blot of brain hippocampus tissue. The results showed that there was no significant difference in the ratio of BDNF to internal control actin among the groups (K=6.856, P=0.144) (P>0.05) (**Figure 7**).

## Discussion

Many studies have been conducted on the relationship between vitamin D and depression, but the conclusions of the studies have not been consistent. For example, there have been human studies on vitamin D levels and the effect of vitamin D rs2228570 (FokI) polymorphism on the etiology and/or severity of diagnosed major depressive disorder, but the results showed no statistically significant differences in vitamin D levels or genotype distribution between groups (23). However, in another study involving a middle-aged group, a prospective association was explored by assessing baseline vitamin D status and depression as measured at follow-up assessment. It was found that, among participants without baseline depression, those with vitamin D insufficiency and VDD were more likely to have new-onset depression. VDD and vitamin D insufficiency may be risk factors of depression in middle-aged people, and VDD may also be a predictor of persistent depressive symptoms in people who are already depressed (24). It was also found that women with inadequate vitamin D levels (≤20 ng/L) were more likely to report elevated depressive symptoms at follow-up assessment, particularly in high-risk groups for depression, such as perinatal pregnant women, although these findings were not statistically significant. Sleep, anxiety, and underlying vitamin D disturbances in early pregnancy are associated with increased perinatal depression. Therefore, to reduce the risk of perinatal depression, studies have also pointed to interventions including ensuring adequate vitamin D levels during pregnancy as potential therapeutic targets (25). Another systematic review and meta-analysis synthesized evidence from randomized controlled trials comparing reductions in depression in patients treated with vitamin D and those treated with placebo. The findings showed that vitamin D supplementation is significantly better than placebo in reducing depression, adults respond significantly better to vitamin D than children and adolescents, and intermittent high doses of vitamin D orally or a single high dose intramuscularly appear to be more effective than daily oral doses. Studies have also shown that vitamin D supplements are effective and safe for people with depression (26). In sum, studies have shown that vitamin D levels are associated with depression, VDD is a risk factor for depression, and vitamin D supplementation is a potential protection against and treatment option for depression.

In this study, further research was conducted on the prevention of depression in adolescents though establishing an animal depression model. The results of animal behavior tests showed that the depressive behavior of mice was indeed reduced by vitamin D injections, with significant differences among groups (P<0.05). In the blood vitamin D level test, it was found that vitamin D injection did increase blood vitamin D levels. In addition, the blood vitamin D levels of mice decreased after UCMS alone. Whether this is related to stress itself and whether it indicates that a decrease in vitamin D levels may play a mediating role in the occurrence of depressive behavior after stress are questions worth further exploring. Regarding the dose of vitamin D injection, although the medium-dose and high-dose groups had clear advantages in increased vitamin D levels and reduced depressive behavior, whether the corresponding vitamin D dose is the safest and most suitable dose, and whether there are risks such as toxicity, also requires further study.

Some studies in adolescent populations have found that vitamin D status is not associated with depressive symptoms. However, research also highlights that adequate vitamin D levels during adolescence are necessary for a number of other health benefits (27). Therefore, it is possible that vitamin D does not play a substantial role in the occurrence of depression, but there are other influencing factors. In addition, these studies suggest that even if depression is not prevented, vitamin D has other benefits. The present study used an animal model, and there are potential differences between animal and human samples. It is therefore necessary to be cautious when drawing conclusions about how vitamin D affects depressive symptoms in humans and to also conduct relevant studies on humans.

Another question our study explored was whether the effect of vitamin D on depressive symptoms is related to the BDNF pathway. Previous studies, including animal studies such as mouse disease model studies, have confirmed the correlation between the antidepressant pathway of action and BNDF. For example, studies have shown that UCMS aggravated motor dysfunction and depression-like behavior compared to MCAO alone, while calcitriol injection enhanced vitamin D receptor and BDNF expression levels in the hippocampus and improved motor dysfunction and depression-like behavior in PSD model mice. Injection of BDNF-binding protein (TrkB-IgG) almost completely reversed the antidepressant and neuroprotective effects of vitamin D, strongly suggesting that vitamin D improved motor dysfunction and depression-like behavior in PSD model mice by promoting hippocampal BDNF signaling (28). However, the results of the present study showed that there was no significant difference in the ratio of BDNF to internal control actin among the groups. Thus, it was determined that there was no statistical association between depression levels and BDNF protein expression in mice.

Another study constructed an exposure model of other risk factors in mice by establishing a mouse model of pneumoconiosis with anxiety- and depression-like behaviors after 28 days of exposure to coal dust, with vitamin D3 treatment used from the first coal exposure. The results showed that coal dust could increase the expression of hippocampal fibrillary acid protein (GFAP) and the activation of astrocytes and decrease neurogenic differentiation factor 1 (NeuroD1) in the hippocampus, which may be the reason why patients with pneumoconiosis show more anxiety and depression than healthy people. The findings also showed that vitamin D3 significantly alleviated anxiety- and depression-like behaviors, reduced the expression level of GFAP, and increased BDNF and neuronal protection by inhibiting the overactivation of astrocytes. This is also the first evidence that vitamin D may be a new way to treat mood disorders caused by particulate matter (29). In these previous studies, the pathway of the protective effects of vitamin D against depression was seen in the involvement of BDNF. However, in the present study, vitamin D showed effects on preventing and improving depression, no evidence was found that these effects were related to the expression of BDNF protein levels.

However, some limitations of the present study should be acknowledged. First, for the study design, due to the young age of the mice, in order to reduce the sample loss caused by behavioral testing, the behavioral indicators were not tested at the beginning of the experiment, and the baseline of behavioral indicators was lacking, so there was no behavioral control before and after for the same group, which may affect the research conclusions. Second, there were significant differences in the behavioral indicators among the groups of mice, along with notable differences in blood vitamin D levels after the experiment. However, confirming an exact correlation between depressive behavior and vitamin D levels, as well as identifying potential related factors, requires further research. Additionally, it was found that the BDNF protein content across different groups was not as expected, and this conclusion also needs to be further confirmed, with further exploration of whether there are other ways vitamin D may affect depressive behavior.

Furthermore, in this research, only male mice were studied. Female mice and the effects of vitamin D on offspring depression were not studied, and effects of other nutrients on depressive behaviors, previously investigated in other studies, were not explored in this research (30). At the same time, some studies have also investigated the effect of vitamin D supplementation before pregnancy on the cognitive function of the offspring of advanced maternal age (AMA) mice. Vitamin D supplementation was found to promote the development of offspring in AMA mice. Vitamin D supplementation could prevent impaired learning and memory ability in offspring born to AMA mice, with a significant impact on cognitive function in offspring (31). Therefore, the benefits of vitamin D for offspring have been well established in animal models. Although in this study, we conducted a controlled study on mice starting at 4 weeks of age, we did not study the offspring, did not examine cognition and other related functions, and did not investigate the effect of cognitive changes on depression. In order to get closer period to adolescence, 4 weeks old mice were selected. But due to the experimental operation, the death occurred in mice of individual groups, less than 2 ones, which may have an impact on the final result, although through a rigorous statistical analysis. These need to be studied in depth and on a larger scale in the future.

In addition, BDNF has been studied in the past as part of the mechanism of vitamin D’s effect on depression, but the pathways of specific mechanisms, such as the specific signaling involved, have not been determined. For example, vitamin D3 has been found to be effective in producing an antidepressant-like response in mice, which may be related to BDNF/TrkB-related synaptic protein synapsin, and there may be some differential effects in different brain regions, with vitamin D3 increasing BDNF levels in the hippocampus and prefrontal cortex in mice (32). However, in this study, the results showed no significant difference among the groups, and no further in-depth study of BNDF-related pathways was conducted. Supplementary studies were carried out on other substance signaling pathways, which are also pending further in-depth research.

Finally, previous studies of other psychiatric disorders, such as attention deficit hyperactivity disorder and autism spectrum disorder (33), have found significant benefits of vitamin D in children with autism (34). Therefore, for children and adolescents, in order to ensure the safety of vitamin D, more studies on other mental disorders should be conducted to discover the different mechanism characteristics of vitamin D.

## Conclusion

Vitamin D can affect the occurrence and severity of depressive symptoms, and in mouse animal models, vitamin D supplementation can reduce the occurrence of stress-induced depression. No significant association between the mechanism of vitamin D in depression and BDNF protein expression levels has been found.

## Data availability statement

The original contributions presented in the study are included in the article/supplementary materials, further inquiries can be directed to the corresponding author/s.

## Ethics statement

The animal study was approved by the ethics committee of China Medical University. The study was conducted in accordance with the local legislation and institutional requirements.

## Author contributions

XY: Conceptualization, Data curation, Funding acquisition, Methodology, Project administration, Writing – original draft, Writing – review & editing, Investigation. JM: Data curation, Investigation, Methodology, Writing – original draft, Writing – review & editing. YH: Conceptualization, Investigation, Writing – original draft. LL: Conceptualization, Investigation, Methodology, Writing – review & editing. GZ: Data curation, Investigation, Software, Writing – review & editing.
